# The Mediating Role of Meaning in Life in the Effects of Calling on Posttraumatic Stress Symptoms and Growth: A Longitudinal Study of Navy Soldiers Deployed to the Gulf of Aden

**DOI:** 10.3389/fpsyg.2020.599109

**Published:** 2021-01-26

**Authors:** Jeong Hoon Seol, Yonguk Park, Jinsoo Choi, Young Woo Sohn

**Affiliations:** Department of Psychology, Yonsei University, Seoul, South Korea

**Keywords:** deployed soldier, calling, meaning in life, post-traumatic stress, post-traumatic growth

## Abstract

This study examined the mediating role of meaning in life in the effect of calling on posttraumatic stress disorder (PTSD) and posttraumatic growth (PTG) among navy soldiers of the Republic of Korea deployed to the Gulf of Aden, Somalia. Participants responded to the questionnaire survey three times (pre-deployment, deployment, and post-deployment) at 4-month intervals. From the first, second, and third surveys, data were collected for 223, 195, and 103 respondents, respectively. Results showed that calling had a negative effect on PTSD, fully mediated by meaning in life, whereas calling had a positive effect on PTG, partially mediated by meaning in life. Our findings suggest that calling acts as a positive psychological resource for maintaining the meaning in life throughout stressful events experienced during deployment, thereby reducing posttraumatic stress symptoms and promoting post-deployment psychological growth. Finally, theoretical and practical implications and the need for follow-up studies are discussed.

## Introduction

Deployed soldiers who perform assigned tasks in a unique environment confronting the enemy are reported to be at high risk of experiencing extreme stress, and the need for research on their psychological health has increasingly been raised (King et al., 2006; Vogt et al., 2012; Lahav et al., 2017). Many deployed soldiers suffer from psychological maladjustment due to unattended mental health needs at the time of deployment, and they receive or wish to receive psychiatric services in the post-deployment period (Dohrenwend, 2006; Hoge et al., 2007; Pietrzak et al., 2011). Since psychological maladjustment of deployed soldiers is a factor that can negatively affect the survival of the troops, including individual combat power and psychological well-being, the identification of the mechanisms of psychological difficulties they experience and establishment of means for their prevention are essential (Steger et al., 2015).

## Theoretical Background

### Posttraumatic Stress Disorder and Growth

The most common psychological maladjustment disorder among deployed soldiers is posttraumatic stress disorder (PTSD) (Goodson et al., 2011; Larner and Blow, 2011). PTSD is characterized by negative symptoms manifested after being exposed to traumatic events, such as experiencing firsthand a life-threatening event (e.g., war, combat, disaster, or loss of life), witnessing people dying, or responding primarily to such events. Typical symptoms include avoiding irritations related to traumatic experiences, reexperiencing traumatic events, and hypersensitivity (American Psychiatric and Association, 2013; Zhang et al., 2018; Boullion et al., 2020).

Previous research indicates a higher prevalence of PTSD among deployed soldiers than among soldiers with no deployment experience (Ikin et al., 2010; Fulton et al., 2015). Richardson et al. (2010) observed that the prevalence of PTSD among Vietnam and Iraq War veterans was higher than among controls and suffered from PTSD symptoms. In severe cases of PTSD, deployed soldiers experience emotional problems such as depression and anxiety (Cox et al., 2017); behavioral problems such as aggression, alcohol and drug abuse (James et al., 2013); and overall psychological maladjustment and impaired physical functions to the extent of difficulty adapting to military life (Asnaani et al., 2014). In a study on PTSD among Vietnam War veterans, Korean veterans displayed PTSD levels higher than the reference level. Those exposed to combat situations were reported to have a variety of PTSD-related negative physical and psychological symptoms in later years (Kang et al., 2014).

Regardless of country, many soldiers exposed to combat with the deployed region’s enemies suffer from PTSD symptoms, which can hinder troops’ combat capacity and lead to mission failures dealing with human life. Moreover, their post-deployment adaptation to the remaining military service back home is also affected by the earlier exposure to combat situations, which leads to a high turnover rate and overall adverse effects during retirement (Horesh et al., 2011). Further, previous research on traumatic experiences and subsequent psychological adaptation among victims of sexual violence and disasters, including soldiers, indicates that not all traumatic experiences lead to psychological maladjustment such as PTSD and even suggests that personal growth can be gained from traumatic experience (Park et al., 1996).

Posttraumatic growth (PTG) is a positive psychological change that is perceived subjectively following a traumatic event. People with PTG experiences report positive self-perception and changes in interpersonal relationships, new possibilities, and spiritual change (Tedeschi and Calhoun, 1996; Tedeschi et al., 2018). First and foremost, people with traumatic experiences report a positive effect on their mental health and psychological well-being when discovering a meaning leading to overcoming the negative aspects of the traumatic experience (Joseph and Linley, 2006). Conclusively, PTG-induced changes indicate the growth of psychological functions surpassing the pre-trauma level (Maercker and Zoellner, 2004; Park, 2016). From this, it can be assumed that if the pathways leading to PTG among deployed soldiers can be identified, it will further the understanding of this mechanism and enable interventions to improve soldiers’ psychological well-being beyond the level of mere understanding of the PTSD symptoms.

Previous studies on PTG identified a positive interpretation of the meaning in a life-threatening event as a key mechanism for establishing PTG (Park and Ai, 2006; Boullion et al., 2020). Soldiers with traumatic experiences feel distressed when their belief system is challenged. However, they may achieve psychological growth from coping with distress by mobilizing their internal resources and adaptively accepting the trauma experience’s meaning. In particular, discovering and pursuing meaning in life from traumatic experiences is a key factor for deriving individual psychological growth (Owens et al., 2009). To this end, we examined the psychological variables to evaluate levels of PTSD and PTG and identify their predictors among deployed troops at three different points in time: pre-deployment, during deployment, and post-deployment.

### Role of Meaning in Life

People with meaning in life believe that their life has a distinct purpose and meaning and strive intensely to pursue the given meaning (Martela and Steger, 2016). Many studies investigating meaning in life have reported that preserving and pursuing the meaning contribute to personal growth, well-being, and psychological strength building (Steger et al., 2008; Park et al., 2010). A series of studies dedicated to the importance of meaning in life in the course of responding to traumatic experiences have demonstrated a close association between the meaning in life and a mechanism by which psychological difficulties are overcome, and posttraumatic growth takes place (Dursun et al., 2016).

Apart from these studies on the meaning in life among people in general, some studies demonstrate the positive effect of meaning in life among soldiers exposed to combat-related experiences such as war and terrorism. Bryan et al. (2013) observed significant negative correlations between meaning in life and psychological maladjustment variables such as stress and suicide ideation. Owens et al. (2009) also investigated the association between depression and PTSD in multi-war veteran samples. They reported that meaning in life exerted a significant moderating effect on PTSD-induced depression, mitigating the symptoms. Several other studies reported that adaptive meaning-making, including meaning in life, is a significant predictor of PTSD and PTG among veterans (Larner and Blow, 2011; Hijazi et al., 2015; Steger et al., 2015).

### Meaning Making Theory

The meaning-making theory proposed by Park (2010) is a model explaining the psychological adaptation of individuals exposed to traumatic events or stressors. This theory states that a successful meaning-making regarding a traumatic event aids in preserving the meaning in life and overcoming psychological difficulties. According to this model, meaning in life has two components: global meaning and situational meaning. Global meaning is composed of personal beliefs and goals: situational meaning is evoked by an encounter with a stressor. People exposed to a stressor come to perceive the discrepancy between the global meaning they had previously and the situational meaning they perceive on the spur of the moment. If the extent of the discrepancy is large, the meaning of the baseline experience is reconstructed through the meaning-making process. Even in exposure to a traumatic event, successful implementation of meaning-making based on the traumatic experience’s reinterpretation can positively affect the posttraumatic psychological outcome (Steger and Park, 2012; Steger et al., 2015; Kurian et al., 2016; Boullion et al., 2020).

Larner and Blow (2011) also explained the pathways to triggering PTSD or accomplishing PTG in a combat veteran from his traumatic experience in the context of meaning-making coping. They presented a theoretical model that post-deployment PTG can be achieved by performing a positive reinterpretation of the traumatic experience based on the pre-deployment global meaning from a longitudinal view. In our study, calling was selected as the vital resource constituting the global meaning and predictor variable, preserving meaning in life after the traumatic experience, and expediting meaning-making based on the meaning-making theory.

### Calling and Psychological Adjustment

Calling is a sense of purpose based upon which people feel personal satisfaction through their work or make a meaningful social contribution (Dik and Duffy, 2009). People who regard work as their calling value the sense of accomplishment and meaning felt in the process of doing work, rather than material satisfaction, such as economic earnings or performance, and establish their identity through work (Wrzesniewski et al., 1997). Furthermore, Dik et al. (2012) reported that calling is a critical element that helps a person understand the meaning of work in a person’s life. Through this transcendental calling, they realize their duties and roles related to work, fulfill life goals through their work, and perceive their work in tandem with doing public good as their contribution to society.

Many studies have been conducted on calling as a protective factor that can reduce the negative effect perceived in the work domain. Wrzesniewski et al. (1997) revealed that a higher calling is associated with lower levels of depression and job stress and allows for clearer perception of situations and problem-solving strategies in a stressful environment. Likewise, calling lowered anxiety symptoms on duty and appeared as a mechanism to overcome the negative emotions perceived in the military (Park et al., 2018). As examined above, calling is a concept closely associated with stress at work and can act as a protective factor buffering the negative impact of stress. Based on these theoretical underpinnings, it was hypothesized in this study that calling would be a protective factor for PTSD symptoms experienced by post-deployment soldiers.

Further, calling has a positive relationship with life variables such as life meaning and life satisfaction, which also embraces work variables such as occupational identity and job satisfaction (Duffy et al., 2012; Park et al., 2019). The higher the sense of calling regarding work, the more adaptive the work performance, which leads to higher job and life satisfaction (Steger et al., 2010). Moreover, people with a calling express high meaningfulness of work and life based on high self-identity and self-efficacy in occupational activities (Duffy and Sedlacek, 2010; Dobrow and Tosti-Kharas, 2011; Duffy et al., 2012, 2018; Park et al., 2016). Given these previous findings, we devoted our attention to the positive effect of calling on PTG.

### Calling as a Source of Meaning in Life

A closer look at individual occupational calling based on the meaning-making theory can reveal a close association between purposeful work and prosocial orientation factors constituting calling and the belief and goal factors constituting the global meaning (Dik and Duffy, 2009; Park, 2010). Among the factors constituting occupational calling, the purposeful work factor is related to the belief factor in terms of the perception that work experience can bring purpose and meaning to life. In the same vein, the prosocial motivation factor is related to the goal factor in that it refers to an awareness of contributing to the public. Thus, occupational calling is not only an individual belief in one’s job but also a factor related to a goal that exercises a positive influence on society. In this regard, occupational calling can be viewed as closely associated with the global meaning concept. Therefore, the perception of occupational activity through calling can act as a cognitive resource that allows for reinterpretation of the given event in a positive direction during the meaning-making process in a highly stressful experience.

**FIGURE 1:**
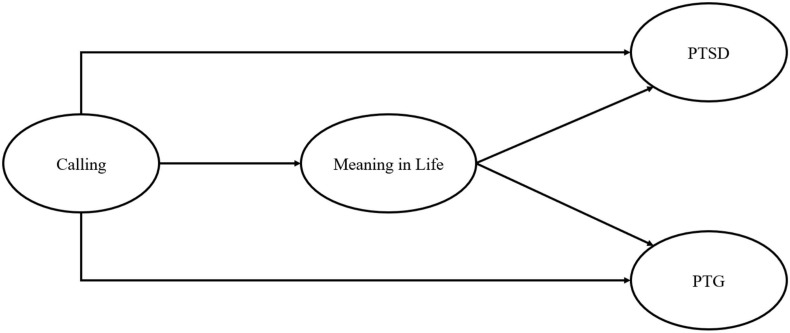
Research model.

Drawing on previous studies, we hypothesized that calling acts as a resource that preserves the meaning in life among deployed soldiers and fosters the meaning-making process and, finally, will predict the post-deployment report of PTSD and PTG significantly. Furthermore, PTSD and PTG were assumed to be significantly related because the coexistence was observed in trauma experiencers (Tedeschi and Calhoun, 1996; Kurian et al., 2016; Zhou et al., 2018). We illustrated the relationships between calling and meaning in life, PTSD, and PTG (see Figure 1) and set these two pathways as hypotheses as follows.

**Hypothesis 1**: Calling has a negative effect on PTSD (H1-a) and a positive effect on PTG (H1-b).

**Hypothesis 2**: Meaning in life mediates the relationships between calling and PTSD (H2-a) and between calling and PTG (H2-b).

## Materials and Methods

### Participants and Procedures

To collect data for the analysis of this study, we administered a questionnaire survey to 223 soldiers belonging to the Navy deployment troops affiliated with the Cheonghae Unit stationed in the Gulf of Aden. The questionnaire was conducted at three different time points over a total of 8 months: pre-deployment (T1) conducted during the researcher’s visit to the corresponding troops; deployment (T2) conducted 4 months later by the duty officer in charge of the questionnaire in coordination with the researcher; and post-deployment (T3) 4 months later conducted during the researcher’s revisit.

Before each survey, the participants were informed of the following: (1) This study was approved by the Ministry of National Defense and the Republic of Korea Navy Headquarters. (2) They have the right to refuse to participate in the study without giving reasons and to stop filling in the questionnaire at any time if they feel the task burdensome. (3) All personal information indicated in the questionnaire is subject to thorough confidentiality in compliance with the Statistics Act and is not reflected in their performance appraisal.

At T1, the respondents answered demographic questions, such as age, gender, marital status, rank, and the measurement of calling the predictor variable, and at T2, they answered questions regarding meaning in life, the mediating variable. At T3, they answered the questionnaires regarding PTSD and PTG, the outcome variables.

The numbers of T1, T2, and T3 respondents were 223, 195, and 103, respectively, and the data from the 103 respondents who participated in all three questionnaire surveys were used for the analysis. The final participants were primarily men (92.2%, *n* = 95), married (82.5%, *n* = 85), and enlisted soldiers (57.7%, *n* = 60). Participants’ age ranged from 20 to 52 years, with an average of 24.78 (*SD* = 6.16).

### Instruments

#### Calling and Vocation Questionnaire

Occupational calling was assessed using the Korean Version of the Calling and Vocation Questionnaire (CVQ-K) based on the CVQ developed by Dik et al. (2012) and validated by Shim and Yoo (2012). The CVQ-K consists of three subdimensions of transcendent summons, purposeful work, and prosocial orientation, with each dimension composed of four items, total 12 items. As proposed in the study in which the original scale was developed, the items were rated on a 4-point Likert scale ranging from 1 (Not at all true of me) to 4 (Absolutely true of me). The higher the total score of the CVQ-K, the higher the perception of calling. Internal consistency reliability for this study was 0.90.

#### Meaning in Life Questionnaire

The Meaning in Life Questionnaire (MLQ) was developed by Steger et al. (2006), and its Korean version was validated by Won et al. (2005). The MLQ consists of two dimensions of meaning in life: presence of meaning and search for meaning. In this study, we used the presence subscale (MLQ-P) to measure how meaningful participants’ lives were during the deployment period. The above scale consists of five questions (e.g., “My life has a clear sense of purpose.” and “I understand my life’s meaning.”). We rated each item on a 7-point Likert scale ranging from 1 (Absolutely untrue) to 7 (Absolutely true), whereby the higher total score is indicative of higher meaning in life of the soldier. Internal consistency reliability for this study was 0.95.

#### Revised Version of the Impact Event Scale

The levels of posttraumatic stress experienced by Cheonghae Unit members were measured with the Revised Korean Version of the Impact Event Scale (IES-R-K), which was revised and validated by Eun et al. (2005) based on the original scale developed by Weiss and Marmar (1997). The IES is widely known as a scale explaining the extent of impact perception following a traumatic experience (Horowitz et al., 1979; Jo et al., 2018). This 22-item questionnaire consists of three clusters of symptoms: hyperarousal, avoidance, and intrusion. We rated each item on a 5-point Likert scale ranging from 0 (not at all) to 4 (extremely), whereby the higher total score is indicative of the higher level of the impact perceived by the soldier.

Additionally, when checked against the PTSD screening cutoff of 24 points and symptom cutoff of 17 points proposed by Eun et al. (2005), 47% of the participants (49/103) satisfied the partial PTSD symptoms. Internal consistency reliability for this study was 0.98.

#### Posttraumatic Growth Inventory

Positive post-deployment changes perceived by Cheonghae Unit members were measured using the Korean Version of the Posttraumatic Growth Inventory (K-PTGI), which was validated by Song et al. (2009) based on the original PTGI developed by Tedeschi and Calhoun (1996). This 16-item inventory consists of four subdimensions: changes in self-awareness, increased depth of interpersonal relationships, discovery of new possibilities, and increased spiritual/religious interest. Each item was rated on a 6-point Likert scale ranging from 0 (strongly disagree) to 5 (strongly agree). The higher the total score of the PTGI, the higher perception of the growth. Internal consistency reliability for this study was 0.95.

### Statistical Analysis

We conducted the descriptive statistics and preliminary analyses using SPSS 25.0 to examine the skewness and kurtosis of data for our study variables based on Weston and Gore (2006). No variables that approached thresholds of recommendations (skewness > | 3 | or kurtosis > | 10 |) were detected.

We used AMOS 20.0 to evaluate our models based on the following model fit indices, chi-square (χ^2^), comparative fit index (CFI), Tucker–Lewis index (TLI), standardized root-mean square residual (SRMR), and root-mean square error of approximation (RMSEA). We examined our model fit based on Hu and Bentler’s (1999) guidelines.

## Results

Preliminary analyses were conducted to assess attrition effects. To assess attrition, we compared the Time 1 data for the longitudinal sample (*n* = 103) who completed the survey at all time points with the Time 1 data for the participants who dropped out at Time 2 or Time 3 (*n* = 120) with a *t*-test. This analysis revealed no statistically significant differences between the longitudinal and attrition group in age [*t*(221) = 1.23, *p* > 0.05], gender [*t*(221) = −0.86, *p* > 0.05], marital status [*t*(221) = 1.36, *p* > 0.05], rank ratio [*t*(221) = 0.26, *p* > 0.05], and calling [*t*(221) = 1.28, *p* > 0.05]. The results suggested that the attrition was not systematic in general for the study variables.

### Descriptive Statistics and Correlation Analysis

Table 1 displays the results of the descriptive statistics and correlation coefficient of our study variables. Our analyses revealed that all our study variables were significantly correlated with each other. To be specific, calling was positively related to meaning in life (*r* = 0.33, *p* < 0.01) and PTG (*r* = 0.45, *p* < 0.01), and negatively related with PTSD (*r* = −0.20, *p* < 0.05). Meaning in life was negatively related with PTSD (*r* = −0.18, *p* < 0.05) and positively related with PTG (*r* = 0.41, *p* < 0.01). Lastly, a negative relationship was found between PTSD and PTG (*r* = −0.37, *p* < 0.01).

**TABLE 1:** Descriptive statistics and correlation analysis.

	M	SD	1	2	3	4
1. Calling (Time 1)	2.64	0.53	−			
2. Meaning in life (Time 2)	5.82	0.97	0.33**	−		
3. PTSD (Time 3)	0.70	0.76	−0.20*	−0.18*	−	
4. PTG (Time 3)	2.98	1.16	0.45**	0.41**	−0.37**	−

*n* = 103, *p < 0.05, **p < 0.01.

### Measurement Model

We conducted confirmatory factor analysis (CFA) to examine the goodness of fit. CFA was used to test the adequacy of our research model’s measurement model before conducting an analysis of the structural model. To evaluated model fit, we used the cutoff criteria of CFI ≥ 0.95, SRMR, and RMSEA ≤ 0.08 (Hu and Bentler, 1999). We conducted CFA four times, respectively, with different measurement models. Our research model which was comprised of 4 factors (calling, meaning in life, PTSD, and PTG) demonstrated the best model fit indices compared with other measurement models. Table 2 displays the results of the CFA of four different measurement models. The measurement model of our research model (4 factors) was a good fit for our data: values χ^2^ (47) = 74.17, *p* < 0.001; CFI = 0.97; TLI = 0.97; RMSEA = 0.07; SRMR = 0.07.

**TABLE 2:** Confirmatory factor analysis results.

Category	χ^2^	*df*	*χ*^2^/*df*	CFI	TLI	RMSEA	SRMR
Model 1 (1 factor)	732.13	53	13.81	0.46	0.33	0.35	0.19
Model 2 (2 factors)	607.62	52	11.68	0.56	0.44	0.32	0.19
Model 3 (3 factors)	503.09	50	10.06	0.64	0.52	0.29	0.16
Research Model (4 factors)	74.17	47	1.57	0.97	0.97	0.07	0.07

**n* = 103, CFI = comparative fit index, TLI = Tucker-Lewis index, RMSEA = root-mean square error of approximation, SRMR = standardized root-mean square residual. Model 1 (1 factor): All items are loaded on one factor. Model 2 (2 factors): T1/2 (calling, meaning in life), T3 (PTSD, PTG). Model 3 (3 factors): T1 (calling), T2 (meaning in life), T3 (PTSD, PTG). Research Model (4 factors): calling, meaning in life, PTSD, PTG.*

### Structural Model

Next, we tested a structural model to examine our hypotheses. The model consisted of one exogenous factor (calling) and three endogenous variables (meaning in life, PTSD, and PTG). Our hypothesized structural model also demonstrated a good fit to our data: χ^2^ (47) = 74.17; CFI = 0.97; TLI = 0.97; SRMR = 0.07; RMSEA = 0.07; 90% confidence interval (CI) [0.04, 0.10].

**FIGURE 2:**
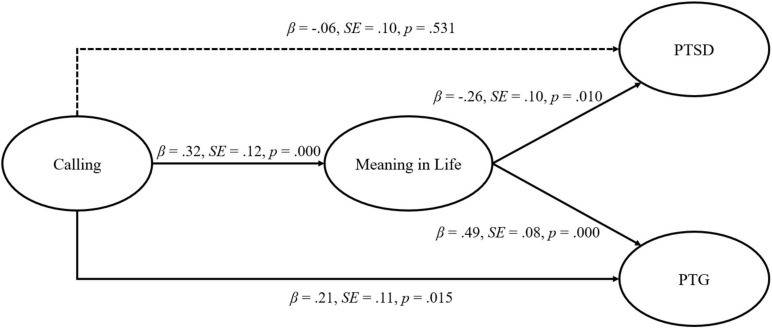
Structural model of calling, meaning in life, PTSD, and PTG.

Figure 2 shows the result indicating that calling had a statistically significant direct effect on meaning in life (β = 0.32, *p* = 0.000) and also that meaning in life had a significant direct effect on PTSD (β = −0.26, *p* = 0.010) and PTG (β = −0.49, *p* = 0.000). In addition, the direct path from calling to PTG was statistically significant (β = 0.21, *p* = 0.015). However, the direct path from calling to PTSD was not significant (β = −0.06, *p* = 0.531). These results support Hypothesis H1-b, but not H1-a.

In the next step, as shown in Table 3, we examined the hypothesized indirect path by using 5000 bootstrapping samples and bias-corrected confidence intervals. The results revealed that calling had statistically significant indirect effects on PTSD (standardized indirect effect = −0.08, *p* = 0.009, BC 95% CI [−0.23, −0.02]) and PTG (standardized indirect effect = 0.16, *p* = 0.009, BC 95% CI [0.04, 0.31]) via meaning in life, supporting Hypothesis H2-a and H2-b. To validate our hypothesis, we employed bootstrapping using a 95% confidence interval, and the result was that no confidence intervals included zero. These reveal the effects of calling on PTSD and PTG mediated by meaning in life.

**TABLE 3:** Standardized indirect path coefficients and bootstrapping test.

	Bootstrapping		BC 95% CI	
Indirect path	Indirect effects	SE	Lower limit	Upper limit
Calling → Meaning in Life → PTSD	−0.08**	0.04	–0.23	–0.02
Calling → Meaning in Life → PTG	0.16**	0.06	0.04	0.31

*n* = 103, bootstrap sample size = 5000, BC 95% CI = bias-corrected 95% confidence intervals. **p < 0.01.

## Discussion

The primary purpose of this study was to verify the mediating effects of meaning in life on the relationship between calling and posttraumatic stress disorder (PTSD) and posttraumatic growth (PTG) among the deployed soldiers belonging to the Republic of Korea Navy Cheonghae Unit. Especially, calling was selected as the core mechanism constituting the global meaning of the meaning-making theory proposed by Park (2010). We theoretically validated the research model in which PTSD symptoms are mitigated, and PTG is fostered through the process that meaning in life is preserved from a stressor and regenerated by calling. To this end, we conducted a questionnaire survey with the soldiers deployed to the Republic of Korea Navy Cheonghae Unit stationed in the Gulf of Aden, Somalia. The questionnaire was administered at three measurement points in time, namely, pre-deployment, mid-deployment, and post-deployment, to clarify the causal relationships between the variables (Larner and Blow, 2011). This approach is expected to improve the psychological health status of deployed service members in theoretical and practical settings, given that it verified the roles of calling and meaning in life that can reduce PTSD symptoms and foster PTG to overcome the traumatic experiences specific to deployed areas.

From the results of this study, the following theoretical implications may be drawn. First, significant correlations were observed between calling, meaning in life, PTSD, and PTG. Calling had a significant positive correlation with meaning in life, supporting the finding of previous studies that calling has a positive effect on life-related variables such as life satisfaction and quality of life (Duffy et al., 2012; Ahn et al., 2019). In particular, it is consistent with previous evidence that calling is a cognitive resource leading to the perception that the work experience is meaningful (Praskova et al., 2014; Thompson and Bunderson, 2019), which contributes to a clear understanding of the meaning in life (Steger and Dik, 2009; Duffy and Sedlacek, 2010).

Additionally, calling had a significant negative correlation with PTSD and a positive correlation with PTG. These results support previous findings that occupational calling acts as a factor capable of protecting people from the negative effects of stressors by reducing psychological maladjustments such as work stress, emotional exhaustion, impaired health status, and sleep problems (Wrzesniewski et al., 1997; Rawat and Nadavulakere, 2015). These results are consistent with reports that calling acts as a core variable contributing to positive adaptation and growth in our work and life (Duffy et al., 2014; Boullion et al., 2020).

Likewise, meaning in life was negatively correlated with PTSD and positively correlated with PTG. These results support those of previous studies that meaning in life is a protective factor for stress-induced PTSD symptoms (Steger et al., 2008) and consistent with the research result of Owens et al. (2009), indicating that meaning in life can reduce the PTSD symptoms caused by the exposure to combat and psychological maladjustment. They are also consistent with observations by Boullion et al. (2020) that meaning in life can become individuals’ cognitive resources as a factor fostering PTG and its role as a protective factor for PTSD.

Lastly, the research model established in this study revealed significant mediating effects of meaning in life in the relationship between calling and PTSD and calling and PTG. These results support the meaning-making model (Park, 2010), suggesting that after a traumatic experience, which is an extremely severe stressor, posttraumatic psychological outcomes can improve as a consequence of a successful meaning restructuring. Furthermore, these are consistent with previous findings that a successful meaning-making can reduce PTSD symptoms and achieve PTG in soldiers exposed to combats (Larner and Blow, 2011; Hijazi et al., 2015).

More specifically, meaning in life partially mediated the relationship between calling and PTG, while the main effect of calling on PTG remained significant. It suggests that calling can act as a core mechanism for realizing PTG. Above all, this indicates the role of calling as a cognitive resource of global meaning in the meaning-making process related to traumatic experiences and concurrently as a direct precipitation factor for PTG. These results are consistent with previous studies (Duffy et al., 2012), which identified calling as the factor eliciting positive psychological outcomes experienced in the job, helping overcome psychological difficulties, and promoting personal growth (Duffy et al., 2014).

On a related note, meaning in life was observed to completely mediate the relationship between calling and PTSD, demonstrating that calling can mitigate PTSD symptoms as a cognitive resource that expedites meaning-making. Interestingly, however, the direct pathway of calling on PTSD was not statistically significant in the research model. Additionally, the correlation analysis revealed a slightly negative correlation between calling and PTSD, which was significant at *r* = −0.20, *p* < 0.05. These results suggest that the main effect of calling on PTSD is lower than that on PTG. For instance, in one study (Jo et al., 2018) on calling and PTSD among Korean firefighters, PTSD was aggravated as calling increased under burnout. These results suggest that calling can paradoxically increase PTSD in certain conditions (Thompson and Bunderson, 2019) and that the relationship between calling and PTSD can be inconsistent. It highlights the need to investigate the relationship between calling and PTSD further, focusing on additional exploration of mediating or moderating variables that may exist between these two variables. This will contribute to deepening the understanding of the relationship between calling and PTSD.

This study examined the role of calling as a factor preventing psychological maladjustment and precipitating psychological growth among deployed soldiers exposed to combat-related traumatic experiences. The following describes the practical implications of this study throughout the deployment process. In selecting soldiers for deployment, it is necessary to consider the degree of occupational calling. At present, only external factors, such as gender, rank, years of service, and family status, are considered when selecting soldiers to be deployed to the Cheonghae Unit stationed in Somalia. Therefore, it is necessary to develop a method to identify individual positive resources such as calling and select soldiers capable of coping with traumatic experiences with adaptive meaning-making.

Moreover, continuous attention and the management system should be provided so that soldiers can hold on to a robust meaning in life system during the deployment service. Personnel management officials should pay attention to the mechanism by which calling can act as a cognitive resource to preserve the meaning in life system, assist with psychological adaptation, and create an environment for managing deployed soldiers who may be suffering from psychological maladjustment.

### Limitation and Future Research

Given that the participants of this study were deployed soldiers of the Republic of Korea Navy unit, which belongs to the Eastern culture, this study has limited generalizability regarding the applicability of the results to their counterparts in the Western culture or the Army or Air Force with different mission areas. Therefore, further research on various samples considering different cultures and military types and examining the psychological changes according to the specific missions of deployed soldiers is warranted.

Despite this limitation, this study is significant because it validates the role of occupational calling in maintaining the meaning in life among deployed soldiers, mitigating PTSD symptoms, and promoting PTG among deployed soldiers and elucidates its importance. Based on continued interest in the psychological health status of deployed soldiers, efforts will be made to assist service members in fulfilling their roles for successful completion of the missions and improving their psychological well-being.

## Data Availability Statement

The datasets presented in this article are not readily available because the release of the raw data should be approved by the Ministry of National Defense and the South Korea Navy Headquarters. Requests to access the datasets should be directed to JS, sr0125@naver.com.

## Ethics Statement

The studies involving human participants were reviewed and approved by the Yonsei University Institutional Review Board. The patients/participants provided their written informed consent to participate in this study.

## Author Contributions

All authors designed the study. JS collected the data, analyzed the model, and led the drafting of the manuscript as a first author. YP and JC consulted on the research design, analyzed the model with JS, and participated in the rewriting of the manuscript. YS supervised the process of this work and repeatedly revised the manuscript. All authors provided critical feedback and approved the final version of the manuscript.

## Conflict of Interest

The authors declare that the research was conducted in the absence of any commercial or financial relationships that could be construed as a potential conflict of interest.
